# Paradise

**Published:** 1889-11-09

**Authors:** 


### Words of Consolation.

### PARADISE.

(*For Reading to the Sick.*)

Solomon, the wisest of men, tells us what our own experience bears out—namely, "that one event happeneth to all men," be he rich or poor, wise or ignorant. Death must come at last to each, and it is both wisdom and satisfaction to find out what the Bible reveals about the state of the departed in the next world. The prophet Isaiah and St. Paul tell us "eye hath not seen nor ear heard neither have entered into the heart of man the things which God hath prepared for them that love Him," and our Lord assured His disciples, "In My Father's house are many mansions; if it were not so I would have told you." But the parable of the rich man and Lazarus in the Gospel of St. Luke gives the fullest account of the matter. In it Christ describes two distinct states, in which the good and the wicked, having been divided immediately after death, wait for the final judgment. These are Hades, the place of torment, and Paradise, the place of happiness, called by the Jews Abraham's bosom, a name which our Lord adopted in this teaching. In it He sets His seal upon the great doctrines, as taught by the Old Testament Church and Scriptures, of the immortality of the soul and of a judgment after death. We will only dwell on Hades for a few moments to notice that the rich man who was condemned to torments does not appear to have been particularly wicked, but simply to have led a careless, self-indulgent life, clad in purple and fine linen, and faring sumptuously every day. He was doubtless selfish and unfeeling, or the beggar would not have been left to the tender mercies of the dogs which licked his sores. A life without love, either to God or man, is hateful to the Creator, and we should take this lesson to heart that punishment is dealt out to those who do not serve him with all their strength. The Paradise to which the beggar was taken, and in which Christ promised the penitent thief that he should meet Him when they had both passed the dark valley of the shadow of death was not Heaven itself, but the near precinct of it. The word Paradise means, enclosed pleasure grounds surrounding a palace. It is the outer court of Heaven, or, as understood by the Jews in the time of our Saviour, "the garden of delight about the palace of God." The repose and refreshment which the redeemed are to enjoy in Paradise are set forth in the Revelations under the image of a flock safely folded by fountains of living waters, and also as eating of the tree of life in the midst of Paradise. However, it is not only rest and refreshment, but action for God's glory, which will occupy the faithful departed. Abraham, we see in the parable, is succouring with fatherly care a once despised servant of God. We may, therefore, expect to meet a happy company of blessed spirits with whom we shall live in glory, doing God's will throughout eternity.

